# Supplementary material for: The causal relationship between the human gut microbiota and pyogenic arthritis: a Mendelian randomization study
Source: Front Cell Infect Microbiol. 2024 Nov 26;14:1452480. doi: 10.3389/fcimb.2024.1452480 (PMC11629706; doi:10.3389/fcimb.2024.1452480)
Supplement: Supplementary file 1 [file DataSheet1.zip › mendelian test/ebi-a-GCST90027493.csv_leave_one_out.pdf]

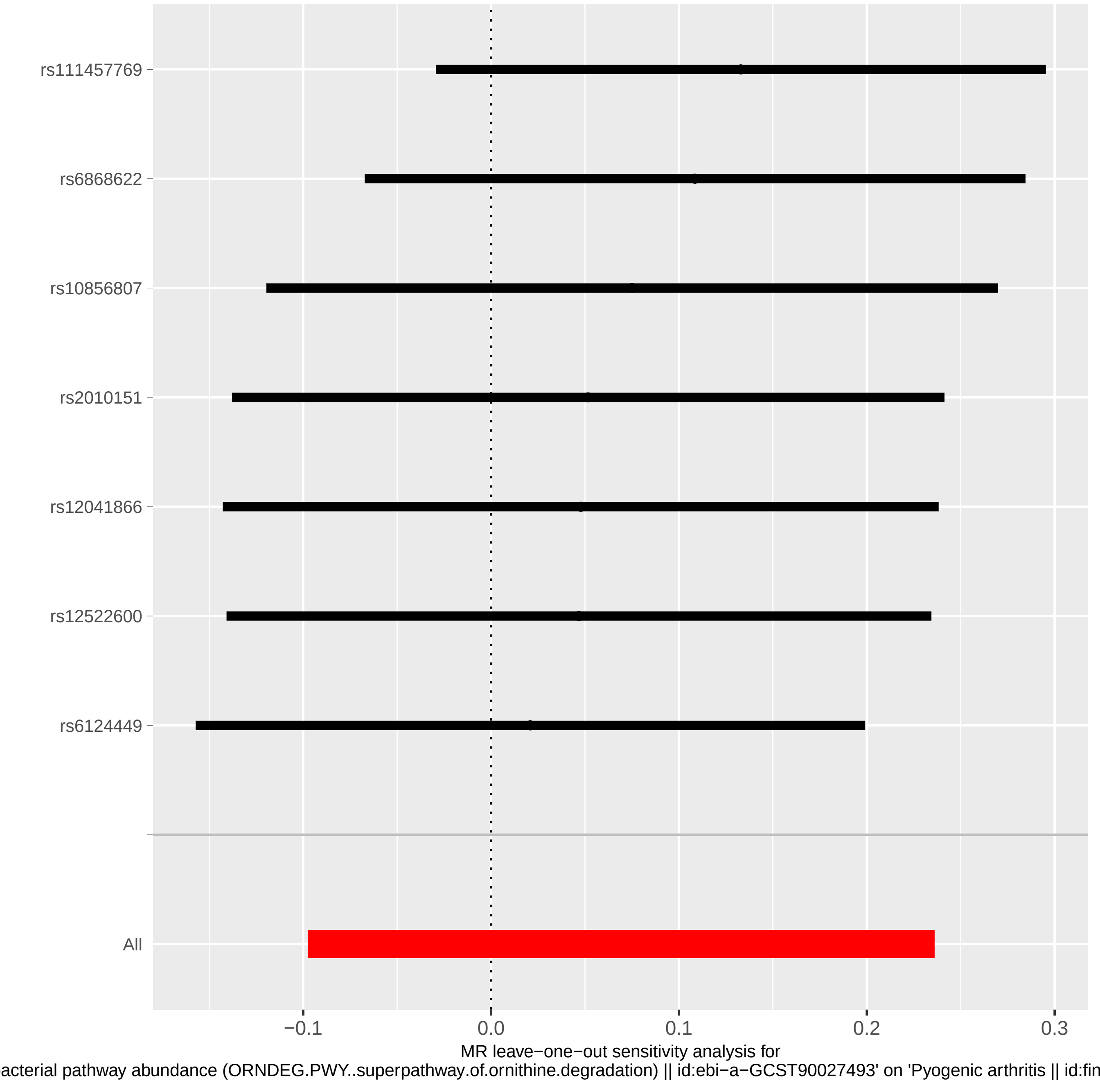

rs111457769
rs6868622
rs10856807
rs2010151
rs12041866
rs12522600
rs6124449
All
−0.1
0.0
0.1
0.2
0.3
MR leave−one−out sensitivity analysis for
acterial pathway abundance (ORNDEG.PWY..superpathway.of.ornithine.degradation) || id:ebi−a−GCST90027493' on 'Pyogenic arthritis || id:fin
